# Presence of HHV-6A in Endometrial Epithelial Cells from Women with Primary Unexplained Infertility

**DOI:** 10.1371/journal.pone.0158304

**Published:** 2016-07-01

**Authors:** Roberto Marci, Valentina Gentili, Daria Bortolotti, Giuseppe Lo Monte, Elisabetta Caselli, Silvia Bolzani, Antonella Rotola, Dario Di Luca, Roberta Rizzo

**Affiliations:** 1 Department of Morphology, Surgery and Experimental Medicine, Section of Gynecology and Obstetrics, University of Ferrara, Ferrara, Italy; 2 School of Medicine, University of Geneve, Geneve, Switzerland; 3 Department of Medical Sciences, Section of Microbiology, University of Ferrara, Ferrara, Italy; 4 Human Reproduction Centre–Brunico Hospital, Brunico (BZ), Italy; Michigan State University, UNITED STATES

## Abstract

To elucidate the roles of human herpesvirus (HHV)-6 primary unexplained infertile women, a prospective randomized study was conducted on a cohort of primary unexplained infertile women and a cohort of control women, with at least one successful pregnancy. HHV-6 DNA was analyzed and the percentage and immune-phenotype of resident endometrial Natural Killer (NK) cells, as the first line of defense towards viral infections, was evaluated in endometrial biopsies. Cytokine levels in uterine flushing samples were analyzed. HHV-6A DNA was found in 43% of endometrial biopsies from primary unexplained infertile women, but not in control women. On the contrary, HHV-6B DNA was absent in endometrial biopsies, but present in PBMCs of both cohorts. Endometrial NK cells presented a different distribution in infertile women with HHV6-A infection compared with infertile women without HHV6-A infection. Notably, we observed a lower percentage of endometrial specific CD56brightCD16- NK cells. We observed an enhanced HHV-6A-specific endometrial NK cell response in HHV-6A positive infertile women, with a marked increase in the number of endometrial NK cells activating towards HHV-6A infected cells. The analysis of uterine flushing samples showed an increase in IL-10 levels and a decrease of IFN-gamma concentrations in infertile women with HHV6-A infection. Our study indicates, for the first time, that HHV-6A infection might be an important factor in female unexplained infertility development, with a possible role in modifying endometrial NK cells immune profile and ability to sustain a successful pregnancy.

## Introduction

HHV-6 is an ubiquitous virus that was first discovered in 1986 [1]. It has been identified as the etiological agent of roseola infantum, and has been implicated (with various degrees) in a number of conditions such as liver disease [2], pneumonitis [3], myocarditis [4], multiple sclerosis [5], drug induced hypersensitivity syndrome [6, 7], the nodular sclerosis subset of Hodgkin’s lymphoma [8], and autoimmune diseases [9]. Since early times after HHV-6 discovery, the existence of the viral variants (HHV-6A and HHV-6B) was recognized [10]. Recently, HHV-6 variants have been recognized as different viral species, on the basis of specific biological, immunological, pathological and molecular characteristics [11].

Although both HHV-6 variants infect mainly T-cells it has wide tropism are important differences in cell tropism between HHV-6A and HHV-6B, HHV-6A but not HHV-6B reproduces in human neural stem cells [12], oligodendrocyte progenitor cells [13] and hepatocytes [14] while HHV-6B infection in astrocytes and hepatocytes result in abortive infection. HHV-6A but not HHV-6B can productively infect CD8+ T cells, natural killer cells and gamma/delta T cells. Some evidence suggests that HHV-6 can also infect and replicate in the human genital tract [15, 16]. In fact: HHV-6 DNA has been detected in genital tract secretions from pregnant and non-pregnant women [17–19]; several studies have reported low-level HHV-6 shedding from the genital tract in up to 25% of women [18–21], with pregnant women characterized by the highest prevalence of shedding [19]; HHV-6 DNA sequences and antigens have been detected in biopsies in archived cervical samples [22–26]. More specifically, the *in-vitro* HHV-6A infection of cervical carcinoma cell lines [27, 28] raises the possibility that the detection HHV-6 footprints reflect the ability of the virus to infect cervical cells, instead of being simply attributed to infected lymphocytes present in the tissue.

These data suggest that the female genital tract may be a secondary site for HHV-6 infection or persistence, although this needs to be confirmed. The possible pathogenic relevance for the genital presence of HHV-6 deserves careful evaluation.

In an attempt to elucidate this understudied aspect of HHV-6 biology, we analyzed the presence of HHV-6 infection in two cohorts of women with differing levels of fertility. Specifically, we studied the prevalence of HHV-6A and HHV-6B infection in the uterine flushing and endometrium biopsies of a randomized group of women with primary infertility group attending an infertility clinic in Italy and a cohort of fertile women. In addition, we assessed the possibility that HHV-6 infection might affect NK cells and cytokine secretion in the uterine environment.

## Materials and Methods

### Clinical Samples

Endometrial tissues were collected from patients that were recruited at admission for tubal patency assessment by Hystero-sono contrast sonography. Inclusion criteria for the study group were: 21–38 years old, regular menstrual cycle (24–35 days), body mass index (BMI) ranging between 18 and 26 Kg/m2, FSH (day 2–3 of the menstrual cycle) <10 mUI/mL, 17-β-Estradiol < 50 pg/ml (day 2–3 of the menstrual cycle), normal karyotype. Women with endometritis, endometriosis, tubal factor, ovulatory dysfunction, anatomical uterine pathologies and recurrent miscarriage were excluded. The stage of the menstrual cycle was categorized into secretory (days 14–28). Five women were enrolled for the collection of endometrial samples in three time points of the mestrual cycle: proliferative (days 5–14), ovulatory (days 11–21) and secretory (days 14–28) phases. Tissue samples collected in HEPES-buffered Dulbecco modified Eagle medium/ Hams F-12 (DMEM/F-12; Invitrogen, Carlsbad, CA) with 1% antibiotic- antimycotic solution (final concentrations: 100 μg/ml penicillin G sodium, 100 μg/ml streptomycin sulfate, 0.25 μg/ml amphotericin B; Invitrogen), and 5% newborn calf serum (NCS; CSL Ltd., Parkville, VIC, Australia), stored at 4°C, and processed within 2 hrs.

### Ethics Statement

Informed written consent was obtained from each patient and ethics approval was obtained from the Ferrara Ethics Committee.

### Preparation of Endometrial Epithelial and Stromal Cells

The endometrium was dissociated in Ca2+ and Mg2+ free phosphate buffered saline (PBS, pH 7.4) containing 300 μg/ml collagenase type III (Worthington Biochemical Corporation, Freehold, NJ) and 40 μg/ml deoxyribonuclease type I (Roche Diagnostics, Mannheim, Germany) in a shaking incubator (Bioline 4700; Edwards Instrument Company, Narellan, NSW, Australia) rotating at 150 rpm at 37°C [29]. At 15-min intervals, the digests were pipetted vigorously and dissociation was monitored microscopically. After 45 min, the cell suspensions were filtered using a 40-μm sieve (Becton Dickinson Labware, Franklin Lakes, NJ) to separate single cells from debris. Further dissociation of the filtrate was prevented by the addition of HEPES-buffered DMEM/F-12/5%FCS. To isolate mononuclear, stromal and epithelial cells we used Ficoll-Paque (Pharmacia Biotechnology, Uppsala, Sweden) and centrifuged for 8–10 min at 390 × *g*. Endometrial epithelial cells were removed from the Ficoll-Paque-medium interface by positive selection using BerEP4-coated magnetic Dynabeads (Dynal Biotech, Oslo, Norway).The beaded epithelial cells were recovered, washed several times in HEPES-buffered DMEM/F-12/1%FCS using a magnetic particle collector (Dynal Biotech) and seeded on basement membrane extract (BME) (Matrigel®, Collaborative Biomedical Products, Bedford, MA, USA) culture plates. The supernatant containing mononuclear and stromal cells were collected and seeded on 100 mm plastic tissue culture dishes. After 12hrs culture the supernatant cells were depleted from the culture, to eliminate mononuclear non adherent cells. Purity of epithelial and stromal components was assessed by morphological determination by light microscopy and reassessed by cytokeratin-18 (CK18) and vimentin staining for epithelial and stromal cells respectively. Each cell population was routinely over 98% purity.

### DNA Analysis

DNA samples were extracted as described [30]. HHV-6 DNA presence and load were analyzed by PCR and real time quantitative (qPCR) specific for the U94 and U42 genes [30], and samples were considered positive when 1 μg of cell DNA harbored more than 100 copies of viral DNA. Amplification of the house-keeping human RNase P or beta-actin genes was used as a control. All clinical samples were analyzed in a randomized and blinded fashion. In addition, when there was enough material to repeat the analysis, the samples were tested again in a randomized and blinded fashion at a distant time from the first analyses. HHV-6 variant A or B identification was obtained by restriction enzyme digestion with HindIII enzyme of the U31 nested PCR amplification product, as reported previously [30]. Digestion products were then visualized on ethidium bromide stained agarose gel after electrophoresis migration.

### Immunofluorescence Assay

Immunofluorescence for HHV-6 antigen expression was performed with a mouse monoclonal antibodies (mAb) directed against p41 and IE2 (early antigen) and glycoprotein gp116 (late antigen) of HHV-6 A and B (ABI, Columbia, MD, USA), as previously described [30]. Epithelial and stromal cells were respectively stained with mouse anti-Cytokeratin- 18, a heterotetramer cytoskeleton protein (CK18) and rabbit anti-vimentin, an intermediate filament of the cytoskeleton proteins moAbs (Abcam, Cambridge, UK), respectively. Nuclei were counterstained with DAPI (4,6-diamidino-2-phenylindole-dihydrochroride) (Sigma-Aldrich, S.Louis, MO, USA).

### Peripheral Blood Mononuclear Cell Purification

Peripheral blood mononuclear cells (PBMC) were isolated from whole blood by Ficoll gradient (Cederlane, Hornby, Ontario, Canada) and resuspended in RPMI medium (EuroClone, Milano, Italy) with 10% FCS, 100U/ml penicillin and 100 U/ml streptomycin (Sigma-Aldrich, S.Louis, MO, USA).

### NK Cell Purification

Peripheral and endometrial NK cells were purified from PBMC and endometrial samples, respectively, through negative magnetic cell separation (MACS) system (Miltenyi Biotech, Gladbach, Germany) [31]. As determined by flow cytometry with CD3-PerCp-Cy5.5, CD56-FITC moAbs (e-Bioscience, Frankfurt, DE), the procedure resulted in >90% pure NK cells (data non shown). Freshly isolated NK cells were cultured for 24 h in medium supplemented with suboptimal doses of IL-12 (1 ng/ml) [31].

### HHV Cell Infection

HeLa cell line (ATCC CCL-2) was grown in DMEM F12 medium (Euroclone) supplemented with L-glutamine in presence of 1% penicillin-streptamycine and 10% of FCS at 37°C with the 5% of CO2. HeLa cell lines were infected with HHV-6A and HSV-1 to evaluate NK cell activation. We used HeLa cells as a good in vitro model of epithelial cells to perform and obtain HSV-1 and HHV-6A in vitro infection.

We used HHV-6A (strain U1102) cell free virus inocula [30] and infected with 10 genome equivalents per 1 cell. We used HSV-1 strain F at a multiplicity of infection of 0.1 PFU (plaque forming unit)/cell for 48 hrs [31]. We used HSV-1 and HHV-6A UV-inactivated viral preparations as controls.

Infected cells were then collected to perform co-culture experiment with NK cells.

### Flow Cytometry

NK cells were defined as CD3-/CD56+ cells. CD158b levels were measured in the CD3-/CD56+ gated cells. Cell viability was assessed by propidium iodide staining. Anti-isotype controls (Exbio, Praha, CZ) were performed. For the CD107a degranulation assay, the cells were incubated 1 h at 37°C with anti-CD107a moAb and treated with Golgi Stop solution (Becton Dickinson, San Jose, CA, USA) for 3 hrs [31]. The CD107a mobilization assay was performed using HHV-6A infected or non-infected HeLa cells as target cells and NK cells from controls or patients as effector cells, with an effector:target ratio of 2:1. 10 ng/ml LPS (Calbiochem, La Jolla, Calif., USA), 25 μg/ml CpG-ODN (ODN-C, TIB MOLBIOL, Genova, Italy) and HSV-1 infected HeLa cells were used as positive control of NK activation. Degranulation was assessed in triplicate after 4 hours of co-culture by staining with PE-Cy5-conjugated anti-CD107a mAb (e-Bioscience, Frankfurt, DE) [31]. Ten thousand events were acquired.

### Statistical Analysis

Statistical analysis was performed with Stat View (SAS Institute Inc). Biological data were analysed by Student T test because they presented a normal distribution (Kruskal Wallis test). Statistical significance was assumed for p<0.05 (two tailed).

## Results

### HHV-6 in Clinical Specimens

We enrolled 30 women with unexplained primary infertility characterized by the absence of previous pregnancies or pathological determinant, and 36 fertile women with at least one previous successful pregnancy. As reported in **Table 1**, no significant differences were present between the two cohorts, except a slight increase in estradiol levels in infertile patients (p = 0.05; Student T test). High estradiol levels could indicate that this hormone is artificially suppressing FSH levels and there could be fertility problems. No differences in progesterone levels were found.

**Table 1:** Women cohorts: demographical and clinical parameters.

Parameters (medain; mean±SD)	Infertile (30)	Control (36)	p value*
Age (yrs)	34.3(31.2±3.2)	34.4(32.5±3.1)	0.98
Duration of infertility (yrs)	3.0(2.7±2.1)	2.1(2.2±1.6)	0.66
Length of menstrual cycle (days)	4.1(3.6±2.4)	4.2(3.9±2.1)	0.45
FSH (mUI/mL) (day 3)	8.7(8.3±2.1)	7.2(7.1±3.1)	0.43
LH (mUI/mL) (day 3)	7.1(6.9±2.1)	6.9(6.5±2.)	0.43
Estradiol (pg/mL) (day 3)	79.9(75.4±65.2)	66.3(56.2±45.2)	0.05**
TSH (uUI/mL)	3.0(2.9±1.2)	2.5(2.4±2.1)	0.46
FT4 (pg/mL)	2.3(2.1±1.4)	2.5(2.2±1.5)	0.89
Progesterone (pg/mL) (day 21)	16.3(14.6±5.7)	14.0(13.6±6.7)	0.56
Smoke habits (%)	20	19.3	0.88
Day (mestrual cycle) of sample collection	14.1(13.9±1.9)	13.8(13.2±1.8)	0.84

*Student T test

** Significant p value

All clinical samples were analyzed for the presence of HHV-6 infection and the results are reported in **Table 2**. We observed the presence of HHV-6B DNA in the 25% and 28% of the PBMC of women with primary infertility and control women, respectively (p = 0.75; Fisher exact test). These results are in agreement with literature data [32], that reported the presence of the HHV-6B variant in a 25–30% of peripheral blood samples. HHV-6A DNA was not detected in PBMC of all the subjects. Surprisingly, the 43% (13/30) of the endometrial epithelial cells from women with primary infertility were positive for HHV-6A DNA, while the cohort of control women did not present HHV-6A viral DNA in their endometrial epithelial cells (p = 5.8x10^-6^; Fisher exact test). HHV-6B DNA was not present in endometrial epithelial cells from either group.

**Table 2:** HHV-6 DNA results in peripheral blood mononuclear cells (PBMC) and endometrial biopsies.

Samples (N)	Infertile (30)	Control (36)	p value*
**HHV6-A**			
Endometrial epithelium	13	0	**5.8x10**^-6^**
Endometrial stroma	0	0	NA
PBMC	0	0	NA
**HHV-6B**			
Endometrial epithelium	0	0	NA
Endometrial stroma	0	0	NA
PBMC	8	10	0,75

*Student T test

** Significant p value

**Fig 1:**
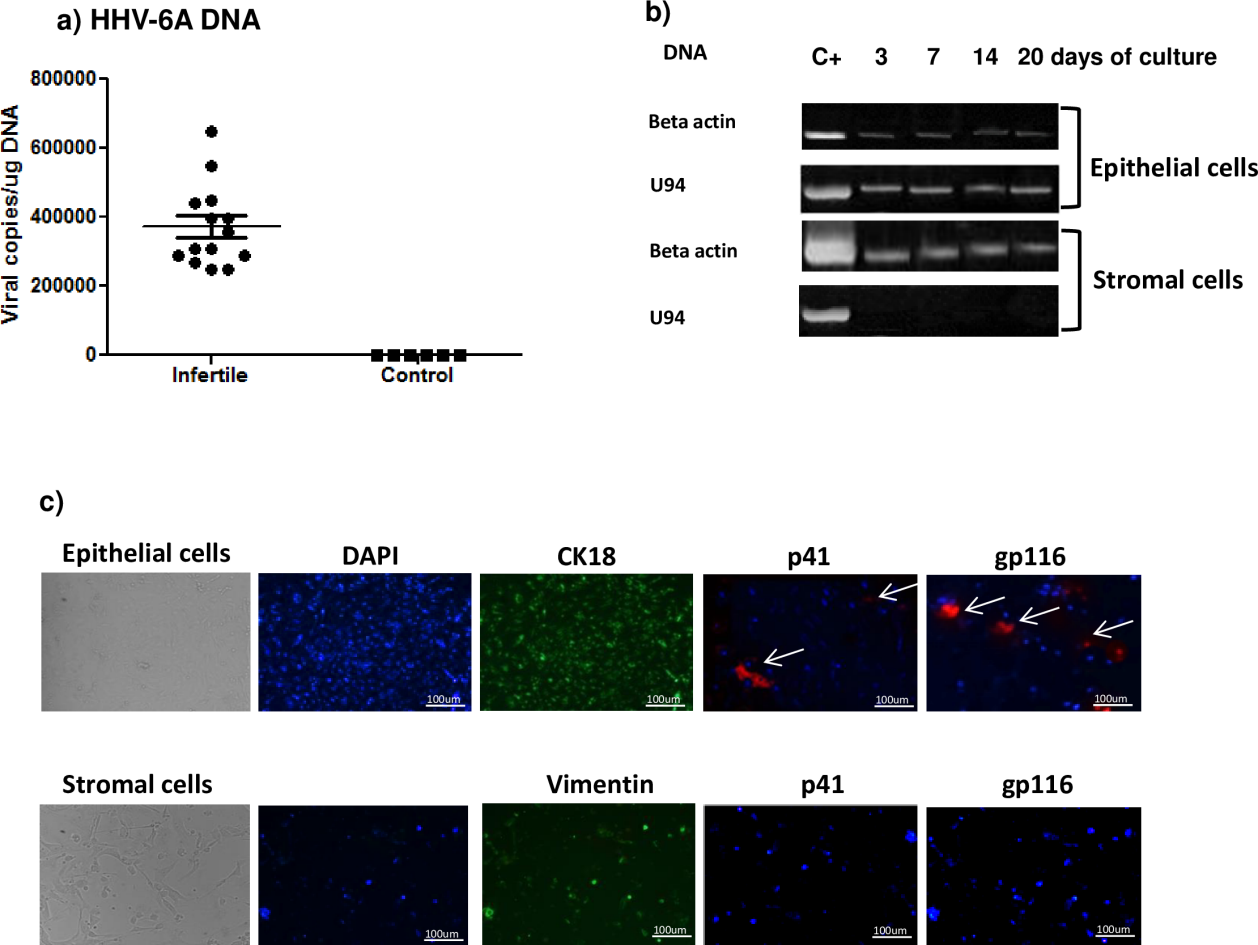
HHV-6 analysis in endometrial biopsies. a) HHV-6 DNA was searched by real time qPCR specific for U42 gene in endometrial biopsies. Results are expressed in viral copies/ug DNA and represent the mean copy number ± SD referred to duplicates of 2 independent assays. Infertile: primary unexplained infertile women; Control: women with at least one successful pregnancy. b) Cell fractions derived by immunomagnetic separation were characterized by PCR specific for HHV-6A U94 DNA after 3, 7, 14 and 20 days of culture. C+: HHV-6A infected J-Jahn cells. c) HHV-6A p41 (early protein) and gp116 (late protein) immunofluorescence (PE) (white arrow) in epithelial and stroma cells from endometrial biopsies of primary infertile women. CK18 and vimentin staining was used to confirm epithelial and stromal cell types, respectively. DAPI staining indicated DNA. Images were taken in bright field (*left panels*) or fluorescence (*right panels*) (Nikon Eclipse TE2000S) equipped with a digital camera. Original magnification 100×.

Endometrial epithelial cells from HHV6-A positive primary infertile women showed an average viral load of 450.000 copies/ug of cellular DNA (range 670.000–250.000 copies/ug DNA), corresponding to about 4 copies of viral DNA per diploid cell (**Fig 1A**). Also stromal cells obtained from the purification of epithelial cells were analyzed for HHV-6 presence and, interestingly, no HHV-6 DNA was detected (**Table 2; Fig 1B**), supporting a localized site of infection for HHV-6A into endometrial epithelium of primary unexplained infertile women.

To be sure of the specific HHV-6A infection in endometrial epithelial cells, we cultured both epithelial and stromal cells and evaluated the presence of HHV-6A proteins. The use of CK18 and vimentin staining in immune-fluorescence confirmed the purification of epithelial cells from stromal cells respectively. We observed the expression of HHV-6A p41 (early) and gp116 (late) proteins after 14 days of culture in approximately 15% of the explanted epithelial cells (**Fig 1C**).

**Fig 2:**
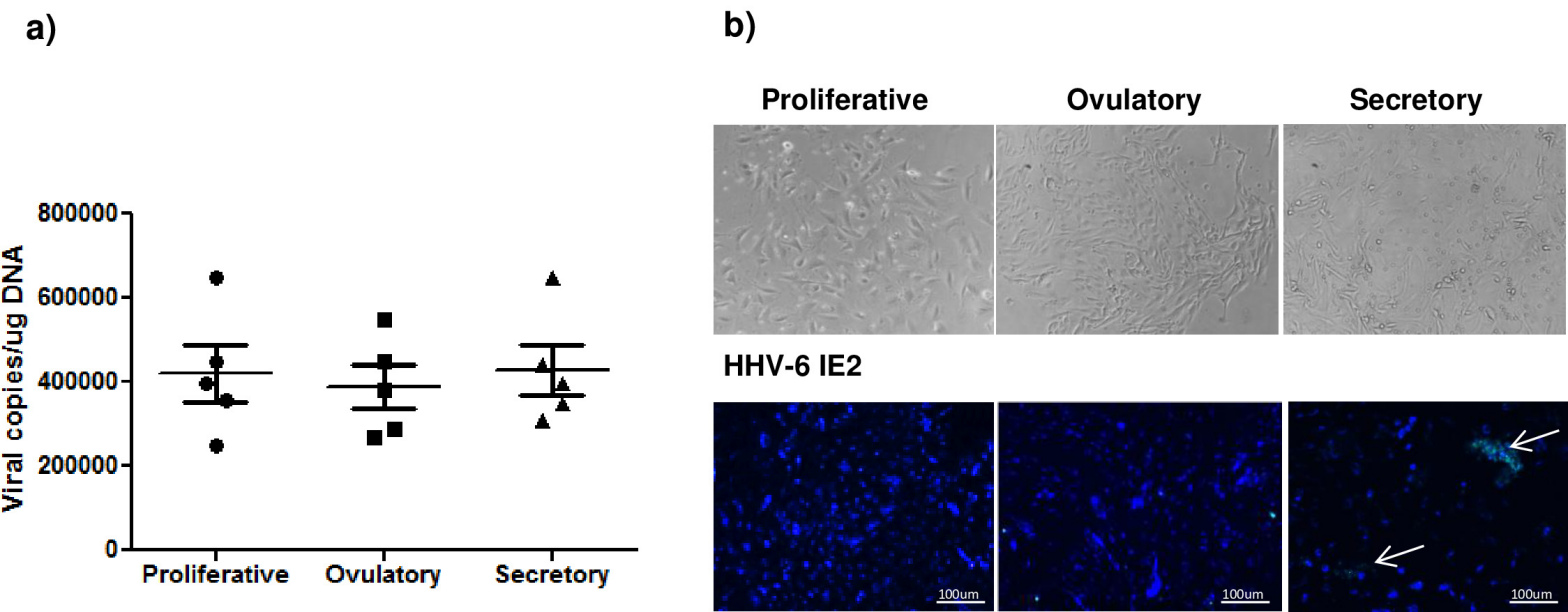
HHV-6A infection and menstrual cycle phases. a) HHV-6 DNA was searched by real time qPCR specific for U42 gene in endometrial biopsies of infertile women during proliferative, ovulatory and secretory phases. Results are expressed in viral copies/ug DNA and represent the mean copy number ± SD referred to duplicates of 2 independent assays. b) HHV-6A IE2 (early protein) immunofluorescence (FITC) (white arrow) in epithelial cells from endometrial biopsies of infertile women during proliferative, ovulatory and secretory phases. DAPI staining indicated DNA. Images were taken in bright field (*left panels*) or fluorescence (*right panels*) (Nikon Eclipse TE2000S) equipped with a digital camera. Original magnification 100×.

Since we performed all the experiments on endometrial cells derived from the secretory phase of the menstrual cycle, we analyzed HHV-6A protein expression by endometrial cells also from proliferative and ovulatory phases. As reported in **Fig 2A**, we observed HHV-6A DNA in all three menstrual cycle phases while the expression of HHV-6A IE2 early protein is evident only in the secretory phase (**Fig 2B**), suggesting a sub-clinical reactivation of HHV-6A latent infection during this specific menstrual cycle phase.

### HHV-6A Modifies Endometrial eNK Cell Immune-Phenotype and Uterine Cytokine Environment

Due to the high prevalence of HHV-6A active infection in a percentage of infertile women, we searched for the presence of differences in clinical and immunological parameters, subdividing infertile women on the basis of the presence/absence of HHV-6A infection (**Tables 3 and 4**). Estradiol levels are higher in infertile women with HHV-6A infection in comparison with those HHV-6A negative (p = 0.045) (**Table 3**). The previous identification of a correlation between estrogen and herpes simplex virus (HSV) reactivation from latency [33] suggests a possible implication of estradiol high levels in HHV-6A infection. In fact, we observed a correlation between the levels of estradiol and the presence of HHV-6A infection (p = 0.02; r^2^: 0.84; Spearman correlation). The other clinical parameters did not present differences in the two cohorts (**Table 3**). When we considered the immunological characteristics, we observed a difference in endometrial (e)NK cell immune-phenotype and cytokine levels in the uterine environment. We found a lower CD56^pos^CD16^neg^ eNK cell number in HHV-6A positive infertile women (65.1 ± 12.1) in comparison with HHV-6A negative infertile women (192.1 ± 28.4) (p = 0.001) (**Table 4**). The distribution of CD56^bright^ and CD56^dim^ eNK cells was different between HHV-6A positive and negative infertile women, with lower CD56^bright^ and CD56^dim^ eNK cells number in HHV-6A positive infertile women (30.0± 15.1; 35.8 ± 10.2) in comparison with HHV-6A negative infertile women (110.3 ± 2.1; 82.1 ± 6.4) (p = 0.001) (**Table 4**). CD3^+^ lymphocytes were not detected in both cohorts, while CD14+ monocytes presented no differences in cell number (p = 0.51). The uterine flushing levels of cytokines showed a different pattern in HHV-6A positive and negative infertile women. We observed a significant increase in IL-10 and a decrease in IFN-gamma levels in HHV-6A positive in comparison with HHV-6A negative infertile women (p = 0.014, p = 0.012, respectively) (**Table 4**). The other cytokines, TNF-alpha, IL-12 and IL-22, were similarly expressed in the uterine flushing samples of the two cohorts (**Table 4**). When we compared infertile and control women, we observed no differences in clinical parameters except for estradiol levels, that are higher in both HHV-6A negative and positive infertile women in comparison with controls (p = 0.042; 0.046, respectively) (**Table 3**). We found a similar frequency of eNK cell subtypes and cytokine levels with HHV-6A negative infertile women and controls (**Table 4**), while HHV-6A positive infertile women presented lower CD56^bright^ and CD56^dim^ eNK cell number, higher IL-10 and lower IFN-gamma levels in uterine flushing samples (**Table 4**). Peripheral blood NK cell subtypes and cytokine levels did not differ between the three cohorts (**S1 Table**).

**Table 3:** Infertile women subdivided on the basis of the presence/absence of HHV-6A infection. Demographical and clinical parameters of infertile and control women.

Parameters (median;mean±SD)	HHV-6A positive	HHV-6 negative	p value*	Control (36)
Age (yrs)	34.3(32.3±2.3)	34.1(33.1±1.5)	0.97	34.4(32.5±3.1)
Duration of infertility (yrs)	3.3(3.1±1.2)	2.1(2.3±2.1)	0.56	2.1(2.2±1.6)
Length of menstrual cycle (days)	3.9(3.8±2.1)	4.4(4.1±1.3)	0.45	4.2(3.9±2.1)
FSH (mUI/mL) (day 3)	9.7(8.9±2.1)	7.0(7.0±3.5)	0.32	7.2(7.1±3.1)
LH (mUI/mL) (day 3)	7.5(7.1±1.3)	6.3(6.1±2.1)	0.45	6.9(6.5±2.)
Estradiol (pg/mL) (day 3)	103.9(99.2±45.2)	72.3(69.6±39.4)	0.045**	66.3(56.2±45.2)
TSH (uUI/mL)	3.2(2.6±2.8)	2.5(2.3±1.4)	0.76	2.5(2.4±2.1)
FT4 (pg/mL)	2.7(2.1±2.5)	2.4(2.2±2.8)	0.89	2.5(2.2±1.5)
Progesterone (pg/mL) (day 21)	17.7(15.2±3.1)	12.0(11.1±4.5)	0.34	14.0(13.6±6.7)
Smoke habits (%)	22	21.3	0.86	19.3
Day (mestrual cycle) of sample collection	14.0(13.1±2.1)	12.9(12.3±1.3)	0.85	13.8(13.2±1.8)

*Student T test

** Significant p value

**Table 4:** Infertile women subdivided on the basis of the presence/absence of HHV-6A infection. Immunological parameters in endometrial samples.

**Immune cells (mean±SD)**	**HHV-6A positive**	**HHV-6 negative**	p value*	**Control**
NK CD56^pos^CD16^neg^ (N)	65.1 ± 12.1	192.1 ± 28.4	0.001**	199.8 ± 21.3
NK CD56^bright^CD16^neg^ (N)	30.0 ± 15.1	110.3 ± 2.1	0.001**	120.1 ± 15.8
NK CD56^dim^CD16^-^ (N)	35.8 ± 10.2	82.1 ± 6.4	0.02**	79.6 ± 5.9
CD3^+^ (N)	0	0	NA	0
CD14^+^ (N)	3.7 ± 4.8	4.2 ± 5.1	0.51	3.9 ± 5.7
**Cytokines(median)**	**HHV-6A positive**	**HHV-6 negative**	p value*	**Control**
IL-10 (pg/ml)	311.9	171.4	0.014**	151.4
IFN-gamma (pg/ml)	65.0	221.5	0.012**	214.4
TNF-alpha (pg/ml)	7.3	5.6	0.31	6.5
IL-22 (pg/ml)	46.9	33.9	0.22	39.8
IL-12 (pg/ml)	210.6	194.4	0.37	198.7

*Student T test

** Significant p value

### HHV-6A Modifies Endometrial NK Cell Functions

**Fig 3:**
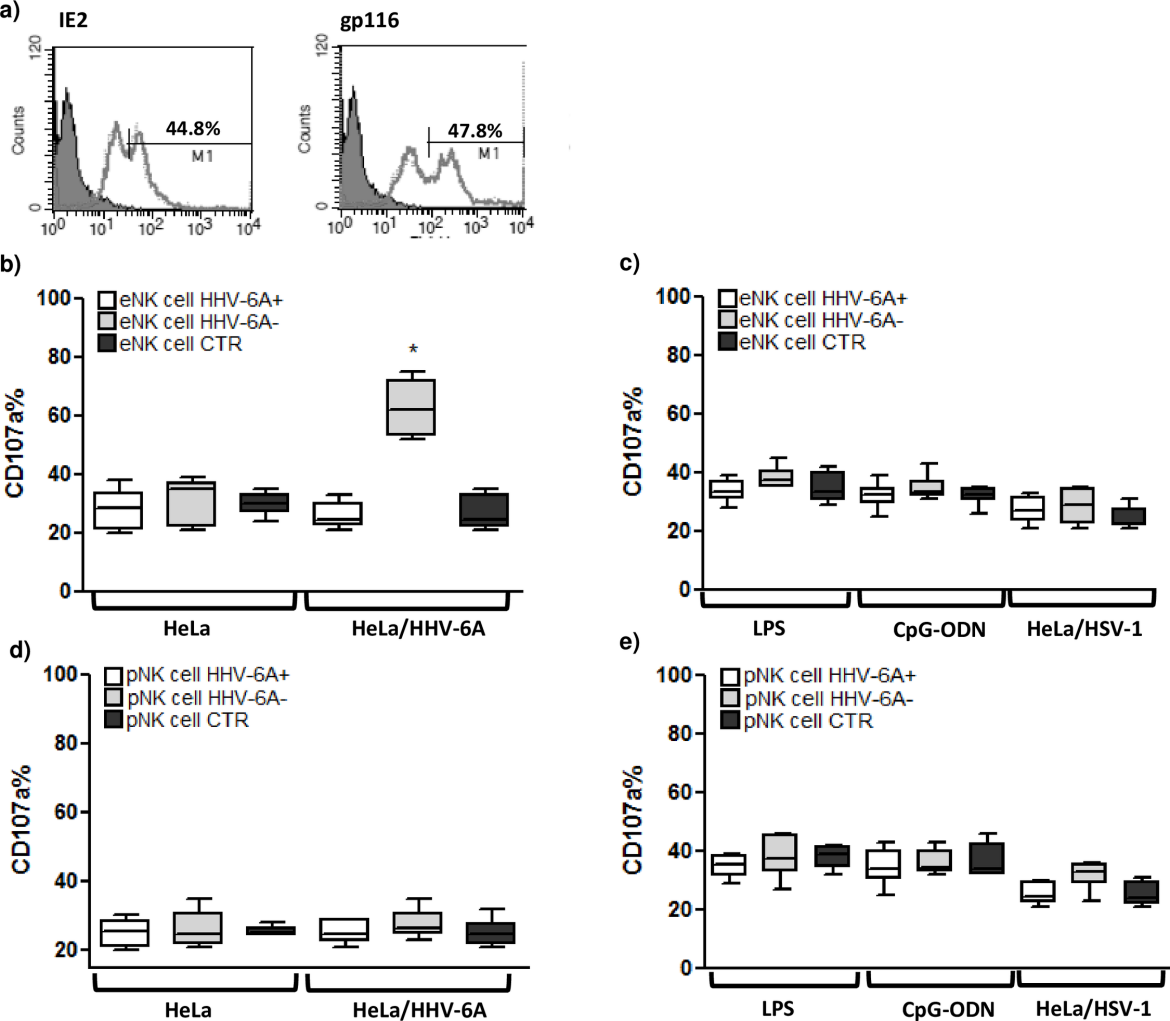
NK cell status. a) HeLa cells were infected with HHV-6A and virus antigen expression was analyzed by flow cytometry using a mouse mAb directed against HHV-6 IE2 early and gp116 late antigens. HeLa HHV-6A infected cells were co-cultured for 4 hours with NK cells, purified from endometrial biopsies and peripheral blood. NK cell activation status was evaluated after CD107a staining by flow cytometry. Percentage of CD107a NK cells are reported after co-culture with: b, d) HHV-6A infected HeLa cells; c, e) LPS, CpG-ODN or HSV-1 infected HeLa cells. Results are expressed in percentage and represent the mean copy number ± SD referred to duplicates of 2 independent assays. eNK cells: endometrial NK cells; pNK cells: peripheral NK cells; HHV-6A+: HHV-6A positive infertile women; HHV-6A-: HHV-6A negative infertile women; CTR: women with a previous successful pregnancy. * significant p value obtained by Student T test.

Immunological memory is classically regarded as an attribute of antigen-specific T and B lymphocytes of the adaptive immune system. Cells of the innate immune system, including NK cells, are assumed to be short-lived cytolytic cells that can rapidly respond against pathogens in an antigen-independent manner and then die off. However, NK cells have recently been described to possess traits of adaptive immunity, such as clonal expansion after viral antigen exposure to generate long-lived memory cells [34]. We evaluated the possible differences between eNK cells response to HHV-6A infection in HHV-6A positive and negative infertile women. As a challenge, we used HeLa, human epithelioid cervix carcinoma, infected with HHV-6A laboratory strain. We observed the expression of HHV-6 proteins on the surface of HeLa cells 3 days post infection (**Fig 3A**). eNK cells purified from endometrial biopsies were challenged with HHV-6A HeLa infected cells and the activation status was evaluated. We observed the highest activation in eNK cells from HHV-6A positive infertile women toward HHV-6A HeLa infected cells (**Fig 3B**). On the contrary, eNK cells from HHV-6A negative infertile women presented a lower activation towards HHV-6A HeLa infected cells (**Fig 3B**), similar to eNK cells from women with a previous successful pregnancy (**Fig 3B**). The same eNK cells were challenged with other stimuli: LPS, CpG-ODN and HSV-1 HeLa infected cells. LPS and CpG-ODN stimulation induced a similar activation in eNK cells from the three cohorts (**Fig 3C**). HSV-1 HeLa infected cells induced a slight increase in CD107a expression in all the three cohorts (**Fig 3C**).

Similarly, peripheral blood pNK cells from the three cohorts were challenged with HHV-6A HeLa infected cells, LPS, CpG-ODN and HSV-1 HeLa infected cells. We observed no activation towards HHV-6A HeLa infected cells (**Fig 3D**) and a slight and comparable increase in the activation of pNK cells from the three cohorts (**Fig 3E**). UV-inactivated viral preparations induced no eNK and pNK cell activation in all the three cohorts (**S1A and S1B Fig**).

## Discussion

Viral infections have been considered as possible environmental factors in human infertility [35]. In particular, herpesviruses have been implicated in male infertility [36], but no specific virus has yet been conclusively identified as associated with female infertility.

In our report, 43% of endometrial epithelial cells from women with unexplained infertility were found positive for HHV-6A DNA, whereas no control women (with at least one previous successful pregnancy) harbored the virus. Furthermore, endometrial epithelial cells from women with unexplained infertility harbored significant viral loads (approximately 4 copies of viral DNA per cell), but no HHV-6A infection was detected in stromal cells and PBMC, excluding the presence of chromosomally integrated HHV-6 DNA [37]. Interestingly, the percentage of HHV-6B positive PBMC in both cohorts was similar to the common population [32]. These observation strengthen the notion that HHV-6A and B have different biological behavior and pathological associations [11]. This different behavior could reflect the fact that HHV-6 variants use different cell receptors. In fact, HHV-6A uses CD46, an ubiquitous molecule present on all cell types [38], while HHV-6B uses CD134, that is expressed mainly on activated Treg T lymphocytes [39]. Furthermore, our results indicate that the uterus of infertile women may constitute a site of active HHV-6A infection/replication. Considering that these women have no HHV-6A in their PBMC, unidentified micro-environmental factors are probably required to allow HHV-6A infection of the uterine epithelial cells. Possibly, the estradiol high levels found in infertile women could act as a co-factor allowing HHV-6 infection of endometrium [33]. Permissiveness to HHV-6A infection of uterine epithelial cells was confirmed by our *ex vivo* experiments showing HHV-6A protein expression during the secretory menstrual cycle phase, suggesting a hormonal implication in HHV-6A sub-clinical reactivation. It is known that steroids cause HHV-6 to replicate disproportionately [40] and reactivate HHV-6 in transplant patients [41]. Here we provide evidence indicating that HHV-6A may induce a modification in eNK cell immune-phenotype and cytokine levels. We observed a decrease in CD56^bright^CD16^neg^ eNK cells in HHV-6A positive infertile women, an increase of Th2 IL-10 cytokine and a decrease of Th1 IFN-gamma cytokine, with an increase of the Th1/Th2 ratio. These results are in agreement with the correlation of Th1/Th2 ratio increase and female infertility condition [42]. Moreover, it is known that HHV-6 infection increases IL-10 expression by monocytes [43] and reduces the release of IFN-gamma [44] by T lymphocytes. Interesting, HHV-6A negative infertile women presented a similar distribution of eNK cell subtypes and cytokine levels in uterine flushing samples in comparison with control women. These data suggest the presence of different immunological components implicated in the infertile condition of the two cohorts of HHV-6A positive and negative women with unexplained infertility. Intriguingly, enhanced HHV-6A-specific eNK cell responses were observed in HHV-6A positive women with unexplained infertility, with a marked increase in the number of eNK cells activating towards HHV-6A infected cells. On the contrary, eNK cells have a similar activation pattern to other stimuli (LPS, CpG-ODN, HSV-1 infection) in comparison with eNK cells from HHV-6A negative women with unexplained infertility and control women. Peripheral blood NK cells from all the three cohorts present no activation towards HHV-6A infected cells and a slight but comparable activation towards LPS, CpG-ODN and HSV-1 infection. These findings are consistent with an abnormal, probably persistent, immune response towards HHV-6A antigens in a subgroup of women with unexplained infertility, possibly favored by the local cytokine and eNK cell environment induced by HHV-6A infection. These HHV-6A-specific responses elicit an over-activation of the eNK cell compartment, as suggested by the increased responses of eNK cells to HHV-6A infection and a different immune-phenotype of eNK cells. In fact, we observed a decrease in CD56^bright^CD16^neg^ eNK cells in HHV-6A positive infertile women, that modifies the eNK cell subset at the uterine environment. These results are in agreement with our previous data on HHV-6 infection in Hashimoto thyroiditis patients, where we observed an increased sensitization of NK cells to this virus [30].

Interestingly, previous observations support our results on the possible adverse effect of HHV-6A on pregnancy: i) Gervasi and coauthors [45] observed the presence of HHV-6 infection in amniotic fluid of a patient that developed gestational hypertension at term and of a patient who delivered at 33 weeks for premature rupture of membranes; ii) Revest and coauthors [46] reported a maternal–fetal HHV-6 infection leading to abortion following HHV-6 seroconversion during pregnancy; iii) Ando and coauthors [47] and Drago and coauthors [48] found a correlation between miscarriages and increased HHV-6 antibody levels and reactivation; Gibson and coauthors [49] observed the association of HHV-6 (and HHV-7, Varicella zoster) newborn infection with preterm pregnancies and pregnancy-induced hypertensive disorders.

However, further studies are required to confirm the association of HHV-6A infection as a trigger of female primary unexplained infertility. Indeed, there are several potential mechanisms by which HHV-6 might induce female infertility. Viral infections might trigger eNK cell functional modifications that could also induce aberrant expression of cytokines thereby promoting a dysfunctional uterine environment.

Overall, our study indicates that HHV-6A infection might be an important factor in female primary unexplained infertility.

## Supporting Information

S1 Fig UV-inactivated viral preparations effect on NK cell status. HeLa cells were infected with UV-inactivated HHV-6A or HSV-1 and co-cultured for 4 hours with NK cells, purified from endometrial biopsies and peripheral blood. NK cell activation status was evaluated after CD107a staining by flow cytometry. After co-culture with UV-inactivated HHV-6A (HeLa/HHV-6A UV) or UV-inactivated HSV-1 (HeLa/HSV-1 UV) infected HeLa cells, we evaluated the percentage of CD107a positive a) endometrial (e)NK and b) peripheral (p)NK cells. Results are expressed in percentage and represent the mean copy number ± SD referred to duplicates of 2 independent assays. eNK cells: endometrial NK cells; pNK cells: peripheral NK cells; HHV-6A-: HHV-6A negative infertile women; CTR: women with a previous successful pregnancy.
(TIF)

S1 Table Immunological parameters of peripheral blood samples. (DOCX)
