# Supplementary figures and images for: Presence of HHV-6A in Endometrial Epithelial Cells from Women with Primary Unexplained Infertility
Source: PLoS One. 2016 Jul 1;11(7):e0158304. doi: 10.1371/journal.pone.0158304 (PMC4930213; doi:10.1371/journal.pone.0158304)

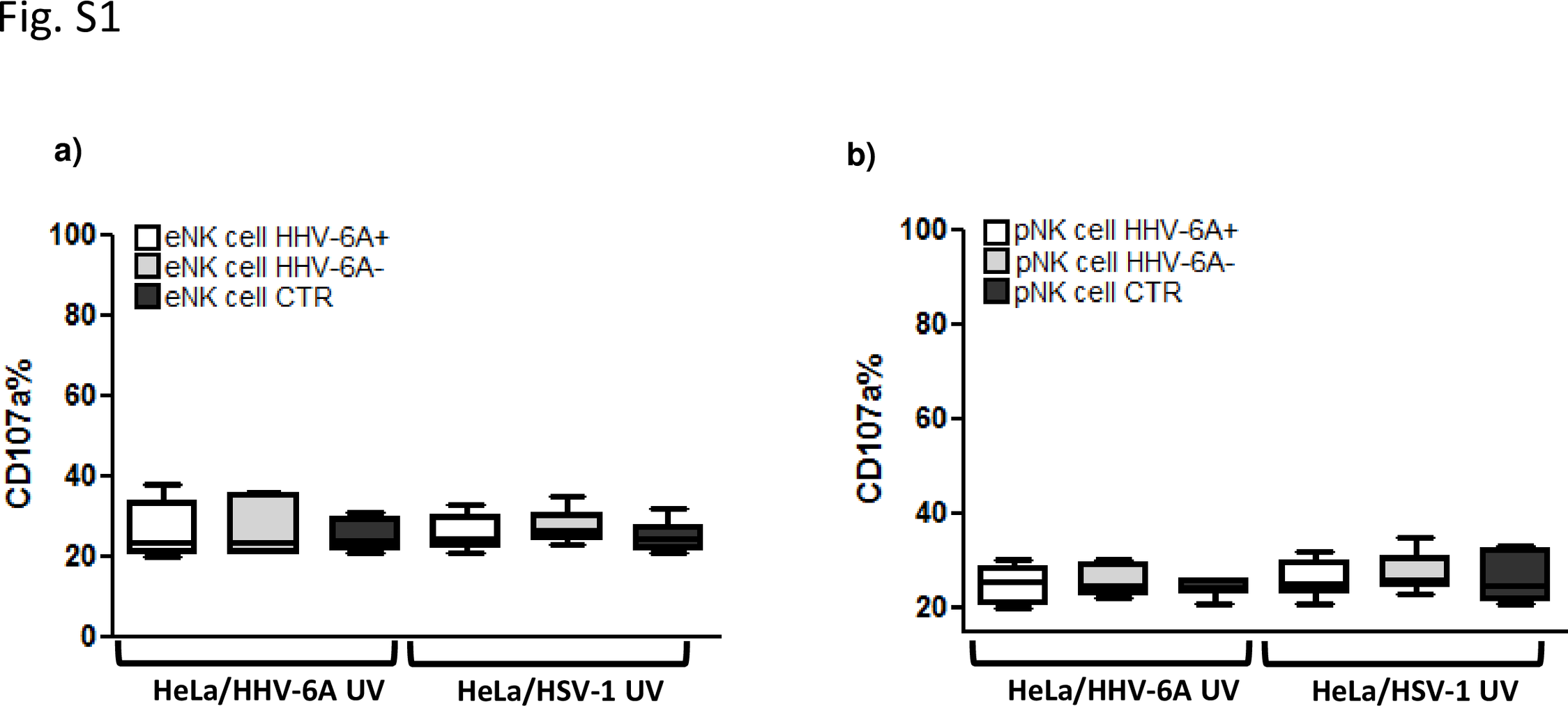

Supplement: S1 Fig — HeLa cells were infected with UV-inactivated HHV-6A or HSV-1 and co-cultured for 4 hours with NK cells, purified from endometrial biopsies and peripheral blood. NK cell activation status was evaluated after CD107a staining by flow cytometry. After co-culture with UV-inactivated HHV-6A (HeLa/HHV-6A UV) or UV-inactivated HSV-1 (HeLa/HSV-1 UV) infected HeLa cells, we evaluated the percentage of CD107a positive a) endometrial (e)NK and b) peripheral (p)NK cells. Results are expressed in percentage and represent the mean copy number ± SD referred to duplicates of 2 independent assays. eNK cells: endometrial NK cells; pNK cells: peripheral NK cells; HHV-6A-: HHV-6A negative infertile women; CTR: women with a previous successful pregnancy. (TIF) [file pone.0158304.s001.tif]
