# Supplementary material for: Presence of HHV-6A in Endometrial Epithelial Cells from Women with Primary Unexplained Infertility
Source: PLoS One. 2016 Jul 1;11(7):e0158304. doi: 10.1371/journal.pone.0158304 (PMC4930213; doi:10.1371/journal.pone.0158304)
Supplement: S1 Table — (DOCX) [file pone.0158304.s002.docx]

**S1 Table. Immunological parameters of peripheral blood samples.**

| **Immune cells (mean±SD)** | **HHV-6A positive** | **HHV-6 negative** | **Control** |
| --- | --- | --- | --- |
| NK CD56^pos^CD16^neg^ (%) | 9.5 ± 3.1 | 9.6 ± 4.4 | 10.3 ± 4.7 |
| NK CD56^bright^CD16^neg^ (%) | 9.0 ± 3.1 | 10.3 ± 2.1 | 9.8 ± 2.8 |
| NK CD56^dim^CD16^-^ (%) | 1.2 ± 1.2 | 1.1 ± 1.4 | 1.3 ± 1.2 |
| CD3^+^ (%) | 71.2 ± 5.1 | 69.8 ± 6.5 | 70.8 ± 4.7 |
| CD14^+^ (%) | 5.7 ± 1.8 | 5.2 ± 2.1 | 6.4 ± 1.5 |
| **Cytokines (median)** | **HHV-6A positive** | **HHV-6 negative** | **Control** |
| IL-10 (pg/ml) | 2.9 ± 3.5 | 3.4 ± 2.3 | 3.3 ± 3.2 |
| IFN-gamma (pg/ml) | 0.8 ± 0.3 | 0.5 ± 0.4 | 0.4 ± 0.5 |
| TNF-alpha (pg/ml) | 13.3 ± 2.9 | 15.6 ± 3.4 | 14.1 ± 3.7 |
| IL-22 (pg/ml) | 12.9 ± 14.2 | 13.9 ± 12.5 | 14.0 ± 13.8 |
| IL-12 (pg/ml) | 23.6 ± 14.2 | 25.4 ± 12.2 | 24.9 ± 13.3 |
